# Effects of *Rht17* in combination with *Vrn-B1* and *Ppd-D1* alleles on agronomic traits in wheat in black earth and non-black earth regions

**DOI:** 10.1186/s12870-020-02514-0

**Published:** 2020-10-14

**Authors:** Pavel Yu. Kroupin, Gennady I. Karlov, Ludmila A. Bespalova, Elena A. Salina, Anastasiya G. Chernook, Nobuyoshi Watanabe, Mikhail S. Bazhenov, Vladimir V. Panchenko, Lubov A. Nazarova, Victor Ya. Kovtunenko, Mikhail G. Divashuk

**Affiliations:** 1 Laboratory of Applied Genomics and Crop Breeding, All-Russia Research Institute of Agricultural Biotechnology, Timiryazevskaya str. 42, Moscow, 127550 Russia; 2 Centre for Molecular Biotechnology, Russian State Agrarian University–Moscow Timiryazev Agricultural Academy, Timiryazevskaya street, 49, Moscow, 127550 Russia; 3 Department of Breeding and Seed Production of Wheat and Triticale, National center of grain named after P.P. Lukyanenko, Central Estate of KNIISH, Krasnodar, 350012 Russia; 4 Kurchatov Genomics Center, Institute of Cytology and Genetics SB RAS, Prospekt Lavrentyeva 10, Novosibirsk, 630090 Russia; 5 College of Agriculture, Ibaraki University, 3-21-1 Chuo, Ami, Inashiki, Ibaraki, 300-0393 Japan; 6 Kurchatov Genomics Center-ARRIAB, All-Russia Research Institute of Agricultural Biotechnology, Timiryazevskaya str. 42, Moscow, 127550 Russia

**Keywords:** Wheat (*Triticum aestivum*), Dwarfing genes, Photoperiod-response genes, Vernalization genes, *Rht*, *Ppd*, *Vrn*, Harvest index

## Abstract

**Background:**

Plant height is an important wheat trait that is regulated by multiple genes, among which *Rht* is of the utmost value. In wheat, *Rht-B1p* (=*Rht17*) is a mutant allele of the *Rht* gene that encodes for a DELLA-protein and results in the development of gibberellin-insensitive plants with a dwarfing phenotype. The pleiotropic effects of dwarfing genes on yield are highly dependent on both the genetic background and the environmental conditions. In Russia, the Central Non-Black Earth Region and Krasnodar Krai are two economically important regions that require differing management for sustainable wheat production for food, feed and industry. The purpose of our study was to compare the pleiotropic effects of *Rht-B1p* on the main valuable agronomic traits in the F_3:4_ families of the spring bread wheat Chris Mutant/Novosibirskaya 67 in the genetic background of *Vrn-B1a*/*vrn-B1* (spring/winter phenotype) and *Ppd-D1a*/*Ppd-D1b* (insensitivity/sensitivity to photoperiod) alleles in a field experiment in Moscow and Krasnodar Krai.

**Results:**

Plant height was reduced on average by 21 cm (28%) and 25 cm (30%), respectively; *Ppd-D1a* slightly strengthened the dwarfing effect in Moscow and mitigated it in Krasnodar Krai. Grain weight of the main spike was reduced by *Rht-B1p* in Moscow and to lesser extent in Krasnodar; *Ppd-D1a* and *Vrn-B1a* tended to partially compensate for this loss in Krasnodar Krai. Thousand grain weight was reduced on average by 5.3 g (16%) and 2.9 g (10%) in Moscow and Krasnodar Krai, respectively, but was partially compensated for by *Ppd-D1a* in Krasnodar Krai. Harvest index was increased due to *Rht-B1p* by 6 and 10% in Moscow and Krasnodar Krai, respectively. *Rht-B1p* resulted in a delay of heading by 1–2 days in Moscow. *Ppd-D1a* accelerated heading by 1 day and 6 days in Moscow and in Krasnodar Krai, respectively.

**Conclusions:**

*Rht-B1p* could be introduced into wheat breeding along with dwarfing genes such as *Rht-B1b* and *Rht-D1b*. Special attention should be paid to its combination with *Ppd-D1a* and *Vrn-B1a* as regulators of developmental rates, compensators of adverse effects of *Rht-B1p* on productivity and enhancers of positive effect of *Rht-B1p* on harvest index.

## Background

Wheat is an important food crop and one of the main sources of calories for humanity. The main factor behind the significant increase in crop yield in the 40th–70th years of the twentieth century, the so-called “Green revolution”, was the development of semi-dwarf wheat varieties (with shorter stature) through the introgression of allelic variants of *Rht* genes, including *Rht-B1b,* which determine plant height and result in a semi-dwarf phenotype. Thanks to the expression of these alleles, it became possible to increase grain yield through better lodging resistance under irrigation and high doses of nitrogen fertilizers, leading to an increase in the number of grains in each spikelet and grain per unit area.

Wild-type alleles *Rht-A1a, Rht-B1a,* and *Rht-D1a* encode DELLA proteins that inhibit the growth of plant cells through negative regulation of the gibberellin signaling pathway, while the degradation of the DELLA protein is promoted by active forms of gibberellins [1]. The phenotypic manifestation of the wild-type allele *Rht-B1a* is a tall plant; presently, at least 15 alleles of this gene are known, of which *Rht-B1c* results in a dwarf phenotype, *Rht-B1b, Rht-B1e, Rht-B1f, Rht-B1h, Rht-B1p* a semi-dwarf phenotype, while plants with *Rht-B1g, Rht-B1i-1* are tall plants (overgrowth mutants) compared to the wild-type allele *Rht-B1a* [2–6]. Alleles *Rht-B1b* and *Rht-B1e* contain a premature stop codon, which is proposed to result in the translation of a form of DELLA protein resistant to proteolysis, and thus permanently inhibiting cell growth [2, 7]. Thus, the dwarfism of plants caused by such mutations of *Rht-1* genes cannot be compensated by the use of exogenous gibberellic acid (GA). Previously we have shown that the dwarfing allele formerly named *Rht17,* now designated *Rht-B1p,* also contains a single-nucleotide substitution that leads to a premature stop codon, so this probably has a similar mechanism of action to other GA-insensitive alleles of *Rht-B1*.

Despite the similarities in the mechanism of action of GA-insensitive alleles of the *Rht-B1* locus, their effect on plant height and distribution in the genetic regions vary considerably [2, 5, 8–10]. An excessively strong decrease in height is caused by *Rht-B1c* (about 50%), which inhibits its widespread use in breeding practice [11, 12]. The most common *Rht-B1b* dwarfing allele leads to a moderate decrease in plant height in the range of 10–25% [13–15]. Locally distributed in Southern Europe is the allele *Rht-B1d*, which reduces height by 10–17% [12, 16]; in East Europe, *Rht-B1e* reduces height by 30% [8, 17, 18]; and in China, *Rht-B1h* produces a 10% height reduction (2). In plants bearing the *Rht-B1p* allele, the effect of plant height reduction is 30–33% in the vegetation experiment in bread wheat [7, 19]. It is known that the inheritance of *Rht-B1p*, like that of the dwarfing alleles with a moderate effect, *Rht-B1b* and *Rht-B1e,* is partially recessive relative to the dominant wild-type allele *Rht-B1a* [7, 20–22].

Dwarfing alleles have an impact not only on plant height, but also have a pleiotropic effect on the timing of plant development and productivity elements. In dwarf plants, assimilate partitioning is improved, making possible the survival of a higher number of florets, thereby increasing the number of grains per spike and improving harvest index [11, 23]. However, GA-insensitivity caused by a number of dwarfing alleles of *Rht-B1* leads to a decrease in the length of the coleoptile from 19 to 27%, that prevents deep seeding in regions with insufficient soil moisture [19, 24–26]; and also to the development of grains with a lower mass [15, 27]. The effect of dwarf plant genes depends on weather and climatic conditions as well as the genetic background [13, 15, 17, 27]. Therefore, it can be expected that the influence of different dwarfing alleles at the *Rht-B1* locus on the elements of productivity will also be different in different growing conditions and in different genetic environments. The effect of *Rht-B1p* on elements of crop structure in spring bread wheat has not been studied and needs to be addressed.

The timing of the onset of such developmental phases as stem elongation, heading and flowering are important adaptive traits of wheat and are controlled by three different signaling pathways: the vernalization (*VRN*), photoperiod (*PPD*), and earliness per se (*EPS*) pathways) [28–30]. Thus, the optimal combination of alleles of these genes plays a main role in the formation of phenological types of plants most suitable for and adapted to agroclimatic regions.

*VRN-1* genes encoding MADS-box transcription factors are involved in the regulation of apical transition from the vegetative to reproductive phase [31, 32]. Of the three homologous genes *VRN-A1, VRN-B1* and *VRN-D1*, located on chromosomes 5A, 5B and 5D respectively, *VRN-A1* has the strongest influence on the phenotype and has an epistatic effect relative to *VRN-B1* and *VRN-D1*. The wild type of the alleles of these genes is associated with a winter growth habit (recessive *vrn-A1, vrn-B1* and *vrn-D1*). Dominant alleles at these loci lead to a spring type growth habit (the transition to the generative phase in such plants occurs without exposure to low positive temperatures) and contain mutations in the promoter and/or the 1st intron relative to alleles of the wild type [33–36]. In regions with cold climates, including Western and Eastern Siberia, for spring wheat with dominant alleles associated with a spring habit (*Vrn-A1a* in combination with *Vrn-B1a* or *Vrn-B1c*), this combination allows the plant to complete its lifecycle after a relatively short vegetative period and escape autumn frosts [37–39]. For southern latitudes, varieties with one allele of *Vrn-B1a* or *Vrn-D1a* can have a definite advantage, as they ripen later, and a longer vegetative period can provide higher yields [37].

In general, spring wheat is a long-day plant. In addition to the *VRN* genes, the timing of heading in wheat is also determined by the allelic condition of the *Ppd* genes regulating the sensitivity of the plant to photoperiod. Furthermore, *Ppd* genes may be regulatory elements for other genes that are involved in light perception (*PhyA*, *PhyB*, *PhyC*) and flowering initiation (*Vrn-1*, *TaFT1*) [40–42]. According to their influence on the rate of plant development, these genes correlate with each other as follows: *Ppd-D1* (strong influence) > *Ppd-B1* > *Ppd-A1* (weak influence) [43–46]. The dominant allele *Ppd-D1a* serves as the main source of insensitivity to photoperiod, which allows plants to flower at the optimal time in a short day. As a result, this allele is common in wheat in southern Europe, where it is exposed to short-day conditions [37, 47]. In regions with long daylight hours, this allele has no significant adaptive advantage and is extremely rare; the *Ppd-D1b* allele is most widespread in such regions [37, 38].

Variation in *Rht, VRN*, and *Ppd* genes can become a tool for the breeder to fine-tune the phenotype for specific agro-ecological conditions, including soil type, temperature, rainfall, and daylight hours. It is known that various allelic variants of *Vrn* and *Ppd* influence not only the duration of wheat phenophases and sensitivity to temperature and light conditions, but also the height of the plant and productivity elements [48–51]. Thus, combining different allelic variants of these genes is a way to develop wheat varieties that are best adapted to the local conditions in terms of straw height, the requirements of temperatures and daylight hours, and the ability to avoid adverse conditions during flowering, heading, ripening and harvesting of grain.

To ensure the economic stability of the Russian regions, it is necessary to develop their own agricultural production, especially wheat, which is supplied both to the domestic and foreign markets for baking, livestock feed, and for starch and alcohol production. The territory of Russia is characterized by a wide variety of agro-climatic conditions. Some regions are more suitable for cultivating winter wheat, others for spring wheat. Spring wheat occupies about half of the crop area under wheat in Russia, and one-third of gross wheat grain harvest. For specific agro-climatic conditions, it is important to choose the combination of straw height and the time of heading/flowering so that the plant can escape stressful conditions in the form of drought or frost, while giving the maximum possible crop of the best quality.

The aim of our work was to study the pleiotropic effect of the *Rht-B1p* allele (in comparison with *Rht-B1a*) on plant height and the main economically significant traits in interaction with the alleles of the vernalization genes *VRN-B1* and sensitivity to photoperiod *Ppd-D1* in spring bread wheat. For field experiments, we selected two regions with contrasting conditions: Moscow and Krasnodar. These areas are characterized by contrasting agro-climatic conditions. The duration of the period with average daily temperatures above 10 °C in Moscow does not exceed 138–140 days, while in the Krasnodar region it lasts for 180–187 days. The soils are sod-podzolic in Moscow and chernozems in Krasnodar. Despite the fact that both regions often receive sufficient and even excessive rainfall per year, the climate of these regions is characterized by sharp fluctuations and unpredictable droughts.

## Results

The allelic state of *Rht-B1*, *Rht-D1*, *Rht-8*, *Ppd-D1*, *VRN-A1*, *VRN-B1*, and *VRN-D1* was revealed via molecular analysis*.* The following genotypes were revealed for the parental plants: Chris Mutant*: Rht-B1p Rht-B1p*\cr*Rht-D1a Rht-D1a*\cr*Rht-8a Rht-8a*\cr*Ppd-D1a Ppd-D1a*\cr*Vrn-A1a Vrn-A1a*\cr*vrn-B1 vrn-B1* \cr*vrn-D1 vrn-D1;* Novosibirskaya 67: *Rht-B1a Rht-B1a*\cr*Rht-D1a Rht-D1a*\cr*Rht-8a Rht-8a*\cr*Ppd-D1b Ppd-D1b*\cr*Vrn-A1a Vrn-A1a*\cr*Vrn-B1a Vrn-B1a*\cr*vrn-D1 vrn-D1.* Therefore, in the F_3:4_ families derived from the cross Chris Mutant/Novosibirskaya 67, segregation in the following three loci occured: *Rht-B1* (*Rht-B1a*/*Rht-B1p*), *Ppd-D1* (*PpdD1a*/*PpdD1b*) and *VRN-B1* (*Vrn-B1a*/*vrn-B1*). For convenience in describing results and comparing values of agronomic traits, we adopted the following abbreviations for the studied alleles:

*Rht-B1a* – R (tall plants, dominant allele).

*Rht-B1p* – r (semi-dwarf plants, recessive allele).

*Vrn-B1а* – V (spring habit, dominant allele).

*vrn-B1* – v (winter habit, recessive allele).

*Ppd-D1a* – P (photoperiod insensitive, dominant allele).

*Ppd-D1b* – p (photoperiod sensitive, recessive allele).

The abbreviations for the genotypes used in the comparison and description of the revealed phenotypic effects are listed in Table 1.

**Table 1:** The abbreviations for the genotypes in families F_3:4_ Chris Mutant/Novosibirskaya 67

Genotype	Abbreviations
*Rht-B1a Rht-B1a Vrn-A1a Vrn-A1a Vrn-B1а Vrn-B1а*	RRVV
*Rht-B1a Rht-B1a Vrn-A1a Vrn-A1a vrn-B1 vrn-B1*	RRvv
*Rht-B1p Rht-B1p Vrn-A1a Vrn-A1a Vrn-B1а Vrn-B1а*	rrVV
*Rht-B1p Rht-B1p Vrn-A1a Vrn-A1a vrn-B1 vrn-B1*	rrvv
*Rht-B1a Rht-B1a Ppd-D1a Ppd-D1a*	RRPP
*Rht-B1a Rht-B1a Ppd-D1b Ppd-D1b*	RRpp
*Rht-B1p Rht-B1p Ppd-D1a Ppd-D1a*	rrPP
*Rht-B1p Rht-B1p Ppd-D1b Ppd-D1b*	rrpp

### Plant height, internode length, and culm weight

**Fig. 1:**
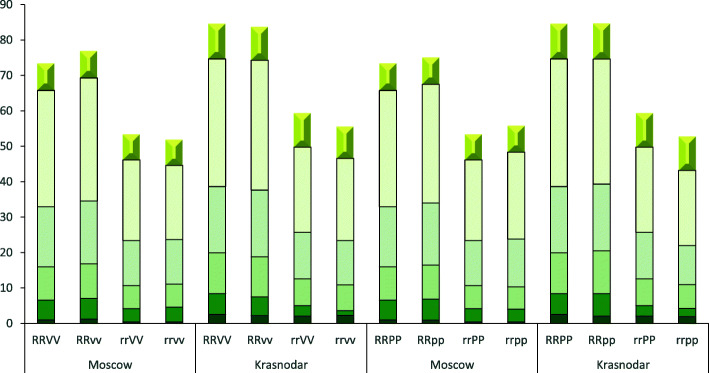
Mean length of spike and internodes in the families F_2:4_ derived from the cross Chris Mutant/Novosibirskaya 67: R – *Rht-B1a*, r – *Rht-B1p*; V – *Vrn-B1a*, v – *vrn-B1*; P – *Ppd-D1a*, p – *Ppd-D1b*. The 4th upper internode is not depicted. Total plant height differs from the mean plant height in Table 4 due to polymorphism in the number of internodes between plants

Both in Moscow and in Krasnodar, a significant difference in height was revealed between plants homozygous for the *Rht-B1p* dwarfing allele (semi-dwarf plants) and plants homozygous for the wild-type allele *Rht-B1a* (tall plants). *Rht-B1p*, compared to the wild-type allele, reduced the height by an average of 21 cm (28%) in Moscow, and by 25 cm (30%) in Krasnodar (Additional file 1 Table S1). At the same time, the difference in height between plants with the RRvv and rrvv genotypes was 25.5 cm (33%), and among families with the spring habit allele *Vrn-B1a* (RRVV and rrVV), was 19.9 cm (27%) in Moscow; and 28.5 cm (34%) and 25.2 cm (30%) in Krasnodar, respectively. The two-way analysis of variance of the height between plants with differing sensitivity to photoperiod demonstrated that the difference in height was 18.9 cm between RRpp and rrpp plants (25%) and 19.9 cm between RRPP and rrPP plants (27%) in Moscow; and 32.6 cm (38%) and 25.2 cm (30%) in Krasnodar, respectively (Table 2, Fig. 1).

**Table 2:** Mean values of biometric traits for main shoot in families F_3:4_ Chris Mutant/Novosibirskaya 67 grouped by *Rht-B1* × *VRN-B1* and *Rht-B1* × *Ppd-D1* alleles

Genotype	Plant height, cm		Peduncle length, cm		Main shoot biomass, g	
	Moscow	Krasnodar	Moscow	Krasnodar	Moscow	Krasnodar
RRVV	73.4 ± 1.5a^a^	84.1 ± 1.0a	32.9 ± 1.1a	36 ± 0.7a	2.3 ± 0.1a	3.6 ± 0.1a
RRvv	77.2 ± 3.3a	83.7 ± 1.5a	34.7 ± 2a	36.6 ± 1.6a	2.5 ± 0.2a	3.7 ± 0.2a
rrVV	53.5 ± 1.4b	58.9 ± 1.2b	22.7 ± 0.9b	24 ± 0.5b	1.8 ± 0.1b	3.1 ± 0.3b
rrvv	51.7 ± 3.9b	55.1 ± 6.4b	20.9 ± 3.1b	23.2 ± 4b	1.7 ± 0.3b	2.4 ± 0.9ab
RRPP	73.4 ± 1.5a	84.1 ± 1a	32.9 ± 1.1a	36 ± 0.7a	2.3 ± 0.1a	3.6 ± 0.1a
RRpp	74.9 ± 1.8a	85.2 ± 1.2a	33.5 ± 1.3a	35.3 ± 0.8a	2.4 ± 0.1a	3.8 ± 0.2a
rrPP	53.5 ± 1.4b	58.9 ± 1.2b	22.7 ± 0.9b	24 ± 0.5b	1.8 ± 0.1b	3.1 ± 0.3b
rrpp	56 ± 2.6b	52.6 ± 1.2c	24.6 ± 1.5b	21.3 ± 0.8c	1.9 ± 0.2b	2.9 ± 0.3b

Mean value± confidence intervals at 0.01 significance level are shown

^a^Mean values designated with the same letters have no significant differences within each group of four genotypes (RRVV, RRvv, rrVV, rrvv and RRPP, RRpp, rrPP, rrpp) as calculated using two-way ANOVA

The decrease in plant height as a whole was due to a significant reduction in the length of each individual internode; however, the length of the peduncle (first upper internode) was most strongly changed. In plants homozygous for the *Rht-B1p* allele, the peduncle was shorter by 10.6 cm (32%) in Moscow and 12.1 cm (34%) in Krasnodar when compared to plants homozygous for *Rht-B1a* grown under the same conditions (Table 2).

In our study, wheat plants had from four to five visible (elongated) internodes on the main stem; while in Moscow, five internodes were found in 37% of tall plants (RR), and 20% of semi-dwarf plants (rr). The 7% variation in plant height in the experiment in Moscow could therefore be explained by the number of internodes. In Krasnodar, five internodes of the main stem accounted for 91% of tall plants (RR), and 63% of semi-dwarf plants (rr). The number of internodes explained 15% of the variability of the plant height in the experiment in Krasnodar.

The presence of the dominant allele of the *Vrn-A1a* gene in all the studied wheat plants means that they all have a spring habit. However, among them, there is segregation of the *VRN-B1* gene, which theoretically can affect the rate of plant development and other agronomically important traits. In our study, families with and without the dominant allele *Vrn-B1a* (contributing to the spring habit), did not differ significantly from each other in plant height in either Moscow or Krasnodar (Additional file 1 Table S1).

Variation in alleles of the *Ppd-D1* gene regulating plant response to day length had a small effect on plant height. In Moscow, *Ppd-D1a* demonstrated the tendency in plant height reduction, whilst in Krasnodar (short daylight hours) it slightly increased height (Additional file 1 Table S1). However, this difference in height did not play a major role in the field experiment. Two-way analysis of variance on the interaction between *Rht-B1* and *Ppd-D1* in Moscow found no significant differences in height between plants. In Krasnodar, in the background of the dwarfing allele *Rht-B1p*, an increase in plant height under the action of an allele insensitive to daylength, *Ppd-D1a,* was 6.3 cm (12%) (Table 2).

Semi-dwarf plant forms had a naturally smaller culm biomass. In plants carrying *Rht-B1p*, compared to plants with *Rht-B1a*, the biomass of the culm on average was 0.5 g less in both regions, which accounted for a 22 and 14% decrease in this trait in Moscow and Krasnodar, respectively. Statistically significant phenotypic differences in the culm biomass between plants with alternate *VRN-B1* alleles were not revealed. In Moscow, *Ppd-D1a* reduced the culm biomass by 0.2 g; however, this effect was not observed after considering particular differences in the two-way analysis of *Rht-B1/Ppd-D1* (Table 2, Additional file 1 Tables S1)*.*

### Structure and productivity of the spike

In a one-way analysis, plants with the *Rht-B1p* dwarfing allele had a shorter and more compact spike compared to tall plants. While plants with the allele of photoperiodic insensitivity, *Ppd-D1a,* had less spike compactness than plants with a genotype sensitive to photoperiod. The relationship between the length and spike compactness and the alleles of the *VRN-B1* gene as a whole does not show a single trend, but rather depends on the growing region and genotype for the *Rht-B1* gene. The strongest influence on spike compactness among the studied genes in Moscow came from *Rht-B1*, while in Krasnodar it was the *Ppd-D1* gene (Additional file 1 Table S5).

Semi-dwarf plants had, on average, lower main spike productivity (grain weight per main spike) both in Moscow (by 0.24 g, 20%) and in Krasnodar (by 0.09 g, 6%) compared to tall plants. Only in the conditions of a short day in the Krasnodar Krai, the allele of photoperiodic insensitivity *Ppd-D1a* contributed to mitigation of the negative effect of *Rht-B1p* on spike productivity (Additional file 1 Table S2). In Krasnodar, in plant families with the photoperiod insensitive allele *Ppd-D1a*, the decrease in spike productivity associated with *Rht-B1p* was 6% (0.08 g), and in the absence of *Ppd-D1a*, it was 15% (0.21 g). In Moscow conditions, *Ppd-D1a* had no effect on spike productivity. Plants differing in alleles of the *VRN-B1* gene did not differ significantly in spike productivity (Table 3).

**Table 3:** Mean values of biometric traits of spike productivity traits in families F_3:4_ Chris Mutant/Novosibirskaya 67 grouped by *Rht-B1* × *VRN-B1* and *Rht-B1* × *Ppd-D1* alleles

Genotype	Grain weight in main spike, g		Grain number per main spike, pcs.		Thousand grain weight, g	
	Moscow	Krasnodar	Moscow	Krasnodar	Moscow	Krasnodar
RRVV	1.17 ± 0.1a^a^	1.43 ± 0.1a	35.2 ± 1.3ab	48.5 ± 1.5a	32.9 ± 0.7a	29.5 ± 0.8a
RRvv	1.25 ± 0.1a	1.51 ± 0.1a	37.7 ± 3a	48.6 ± 3.9a	32.8 ± 1.9a	31.4 ± 1.8a
rrVV	0.95 ± 0.1b	1.35 ± 0.1b	33.6 ± 1.5b	50.2 ± 1.4a	27.6 ± 0.8b	27 ± 0.7b
rrvv	0.9 ± 0.2b	1.07 ± 0.5b	31.4 ± 4.8b	42.6 ± 14a	28.3 ± 1.7b	24.8 ± 5.2b
RRPP	1.17 ± 0.1a	1.43 ± 0.1a	35.2 ± 1.3bc	48.5 ± 1.5a	32.9 ± 0.7a	29.5 ± 0.8a
RRpp	1.24 ± 0.1a	1.41 ± 0.1ab	38.1 ± 1.7a	49.5 ± 1.7a	32.1 ± 1.1a	28.5 ± 0.8a
rrPP	0.95 ± 0.1b	1.35 ± 0.1b	33.6 ± 1.5c	50.2 ± 1.4a	27.6 ± 0.8b	27 ± 0.7b
rrpp	1.01 ± 0.1b	1.2 ± 0.2c	37.5 ± 3.2ab	47.6 ± 3.7a	26.2 ± 1.3b	24.8 ± 2c

Mean value± confidence intervals at 0.01 significance level are shown

^a^Mean values designated with the same letters have no significant differences within each group of four genotypes (RRVV, RRvv, rrVV, rrvv and RRPP, RRpp, rrPP, rrpp) as calculated using two-way ANOVA

The productivity of the ear depends on the number of grains and the average weight of 1000 grains. In Krasnodar, plants with different allelic states of the studied genes *Rht-B1, Ppd-D1*, and *VRN-B1* did not differ significantly in the grain number per spike. In Moscow, allele *Ppd-D1*a reduced the grain number per spike by 3–4 grains (9%) (Additional file 1 Table S2). On average, plants carrying *Vrn-B1a* and *vrn-B1* did not significantly differ in the grain number per spikelet, however, the presence of the dominant allele *Vrn-В1а* mitigated the contrast between short and tall plants, generating a 5% reduction (1–2 kernels) in RRVV vs rrVV (plants with *Vrn-B1a*) compared with a 17% reduction (6 grains) in RRvv vs rrvv (plants with *vrn-B1)*. The influence of the studied alleles on the 1000 grain weight is similar to the above-described influence on the productivity of the spike as a whole. In Moscow, *Rht-B1p* significantly and independently of the allelic condition of the *VRN-B1* and *Ppd-D1* genes, reduces this productivity by 16–18%. But in Krasnodar, the presence of the *Ppd-D1a* allele tended towards mitigation of the negative effect of *Rht-B1p*. Therefore, the dwarfing allele *Rht-B1p* in the absence of the allele *Ppd-D1a* led to a decrease in 1000 grain weight by 13% (RRpp and rrpp), but in the presence of the allele *Ppd-D1a*, 8% only (RRPP and rrPP) (Table 3).

Thus, the alleles insensitive to photoperiod *Ppd-D1a* and *Rht-B1p,* showed in our experiments the opposite effect on 1000 grain weight and grain weight per spike, which allows us to consider *Ppd-D1a* as a compensator for the negative effect of *Rht-B1p* on plant productivity under the conditions of Krasnodar. Moreover, both alleles, *Rht-B1p* and *Ppd-D1a*, showed a tendency to increase the harvest index compared to wild-type alleles *Rht-B1a* and *Ppd-D1b* (Additional file 1 Table S3).

### Heading, the sum of effective temperatures and daylight hours

In Moscow, heading time in semi-dwarf plant families (rr) came slightly later than in tall families (RR) among plants with *Vrn-B1a*. Also, semi-dwarf plants (rr) in families with the *Vrn-B1a* allele required a higher sum of active temperatures (> 10 °C) and active light days (> 12 h) for heading of 5 and 3%, respectively, compared to the tall plants (RR). Under the climatic conditions of Krasnodar, no differences were found between families homozygous for the alleles of the *Rht-B1* and *VRN-B1* genes (Table 4).

**Table 4:** Mean values of heading date, sum of active (> 10 °C) temperatures and active (> 12 h) light days from sowing to heading date in families F_3:4_ Chris Mutant/Novosibirskaya 67 grouped by *Rht-B1* × *VRN-B1* and *Rht-B1* × *Ppd-D1* alleles

Genotype	Heading date, days from sowing to heading		Sum of active (> 10 °C) temperatures to heading date, ^○^C		Sum of active (> 12 h) light days to heading date, h	
	Moscow	Krasnodar	Moscow	Krasnodar	Moscow	Krasnodar
RRVV	52.7 ± 0.7b^a^	65 ± 2.3a	810 ± 15b	861 ± 48a	875 ± 12b	883 ± 35a
RRvv	53.5 ± 1.4ab	62.3 ± 6a	828 ± 30ab	802 ± 132a	890 ± 24ab	841 ± 90a
rrVV	54.4 ± 1a	64.6 ± 1.3a	847 ± 21a	854 ± 28a	906 ± 18a	877 ± 20a
rrvv	54.2 ± 2ab	66 ± 4.1a	842 ± 44ab	884 ± 104a	901 ± 35ab	898 ± 80a
RRPP	52.7 ± 0.7c	65 ± 2.3b	810 ± 15c	861 ± 48b	875 ± 12c	883 ± 35b
RRpp	53.7 ± 0.8bc	71.9 ± 3.7a	831 ± 17bc	1003 ± 76a	893 ± 14bc	988 ± 56a
rrPP	54.4 ± 1ab	64.6 ± 1.3b	847 ± 21ab	854 ± 28b	906 ± 18ab	877 ± 20b
rrpp	55.1 ± 1.2a	70 ± 5.2a	861 ± 25a	967 ± 104a	917 ± 20a	959 ± 80a

Mean value± confidence intervals at 0.01 significance level are shown

^a^Mean values designated with the same letters have no significant differences within each group of four genotypes (RRVV, RRvv, rrVV, rrvv and RRPP, RRpp, rrPP, rrpp) as calculated using two-way ANOVA

In Moscow, the two-way analysis of the interaction of *Rht-B1* and *Ppd-D1* showed that heading in semi-dwarf plants (rr) came a little later compared to tall plants (RR) in families with photoperiod sensitivity allele *Ppd-D1b* and photoperiod insensitivity allele *Ppd-D1a*. The sum of active temperatures and daylight hours are higher for semi-dwarf plants (rr) than tall plants (RR) by an average of 4 and 3%. Tall plants carrying the allele of insensitivity *Ppd-D1a* (RRPP) headed a bit earlier than those with the photoperiod sensitive allele (RRpp), and their demand for the sum of active temperatures and light days decreased by 3 and 2%, respectively (Table 4).

Under the conditions of Krasnodar, we identified a significant difference in heading time between plants homozygous for different alleles of *Ppd-D1*. Families with the *Ppd-D1a* insensitivity allele were earlier than those with the *Ppd-D1b* sensitivity allele, the difference was 7 days among tall plants (RRPP and RRpp), and 5 days among semi-dwarf plants (rrPP and rrpp). Additionally, in the presence of the photoperiod-insensitive allele, *Ppd-D1a*, the demand for active temperatures and light days decreased by an average of 13 and 10%, respectively (Table 4, Additional file 1 Table S4).

### Gene interaction analysis

To identify the most significant relationships between the allelic condition of the studied genes, we conducted a pairwise regression analysis for each pair of genes: *Rht-B1* and *VRN-B1*, *Rht-B1* and *Ppd-D1,* for each region of the field experiment. Correlation coefficients for *Rht-B1* in both pairs of comparison coincided, or were very close, within each region of the field experiment, so we demonstrate here the average value of correlation coefficients (Table 5).

**Table 5:** Correlation coefficients between the presence of the *Rht-B1p*, *Vrn-B1a*, and *Ppd-D1a* alleles in homozygotes and valuable agronomic traits in families F_3:4_ Chris Mutant/Novosibirskaya 67

Agronomic trait	*Rht-B1p*		*Vrn-B1a*		*Ppd-D1a*	
	Moscow	Krasnodar	Moscow	Krasnodar	Moscow	Krasnodar
Plant height (PH)	−0.71^a^	−0.90^a^	−0.03	0.02	−0.06^a^	0.04^a^
Internode number (IN)	−0.13^a^	− 0.27^a^	−0.05	0.02	0.03	−0.15^a^
Peduncle length (PL)	−0.59^a^	− 0.86^a^	− 0.01	− 0.01	− 0.06^a^	0.09^a^
Main spike length (MSL)	− 0.15^a^	− 0.11^a^	− 0.01	0.14^a^	− 0.02	− 0.01
Spikelet number per main spike (SN)	0.003	− 0.05	− 0.04	− 0.02	− 0.15^a^	− 0.26^a^
Spike compactness (SC)	0.16^a^	0.06^a^	− 0.03	− 0.15^a^	− 0.13^a^	−0.23^a^
Main spike weight (MSW)	−0.29^a^	−0.11^a^	− 0.04	−0.01	− 0.09^a^	0.04
Main culm weight (MCW)	−0.54^a^	−0.19^a^	− 0.08^a^	0.004	− 0.10^a^	−0.04
Main shoot biomass (MSB)	−0.40^a^	−0.21^a^	− 0.05	0.00	− 0.10^a^	−0.02
Grain weight per main spike (GW)	−0.29^a^	−0.15^a^	− 0.02	−0.01	− 0.08^a^	0.08^a^
Grain number per main spike (GN)	−0.09^a^	0.06	−0.02	0.04	−0.16^a^	0.01
Grain number per spikelet (GNS)	−0.11^a^	0.09	0.01	0.05	−0.09^a^	0.16^a^
Thousand grain weight, g (W)	−0.45^a^	−0.29^a^	− 0.01	−0.07	0.08^a^	0.14^a^
Harvest index (HI)	0.12^a^	0.07	0.05	0.02	0.02	0.10^a^
Heading date (H)	0.47^a^	−0.06^a^	−0.06	0.19^a^	−0.35^a^	−0.63^a^
Sum of active (> 10 °C) temperatures to heading date, ^○^C (SAT)	0.47^a^	− 0.05^a^	− 0.06	0.19^a^	− 0.36^a^	−0.63^a^
Sum of active (> 12 h) light days to heading date, h (SAD)	0.47^a^	−0.06^a^	− 0.06	0.19^a^	− 0.35^a^	−0.64^a^

^a^correlations significant at the 0.05 significance level

**Fig. 2:**
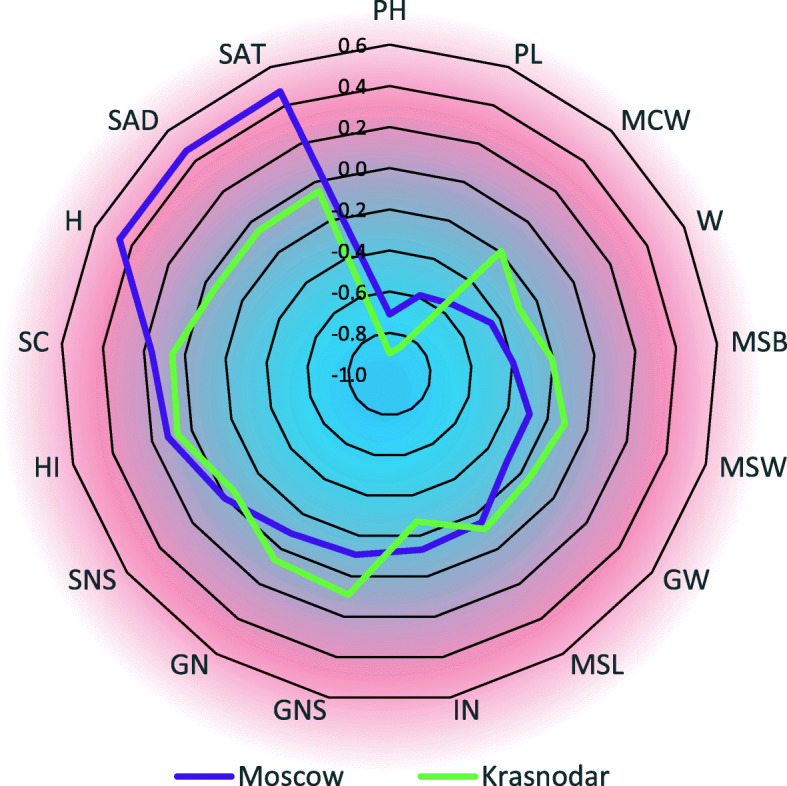
Correlation coefficients between the presence of the *Rht-B1p*, *Vrn-B1a*, and *Ppd-D1a* alleles in homozygotes and valuable agronomic traits in families F_3:4_ Chris Mutant/Novosibirskaya 67 in field tests in Moscow (violet line) and Krasnodar (green line). Blue and red represent negative and positive correlations respectively. PH, plant height; IN, internode number; PL, peduncle length; MSL, main spike length; SN, spikelet number; SC, spike compactness; MSW, main spike weight; MCW, main culm weight; MSB, main shoot biomass; GW, grain weight per main spike; GN, grain number per main spike; GNS, grain number per spikelet; W, thousand grain weight; HI, harvest index; H, heading date; SAT, sum of active (> 10 °C) temperatures to heading date; SAD, sum of active (> 12 h) light days to heading date

In Moscow, a strong negative correlation was shown between the presence of *Rht-B1p* in the genotype and plant height (explaining 50% of the variability). There was a moderate negative correlation between the presence of *Rht-B1p* and main culm weight, length of first internode, 1000 grain weight (20% of the variability), vegetative part of the main shoot, and grain weight of the main spike (8% of the variability). A moderate positive correlation was found between the presence of *Rht-B1p* and the requirements for the sum of active temperatures and active daylight hours, as well as heading date (20% of the variability). For *Ppd-D1a*, a moderate negative correlation with the requirements for the sum of active temperatures, active daylight hours, and heading date (12% of the variability) were revealed (Table 5, Fig. 2).

In Krasnodar, a strong negative correlation was shown between the presence of the *Rht-B1p* in the plant genotype and plant height (explaining 81% of the variability) and the peduncle length; a moderate negative correlation was recorded between the presence of *Rht-B1p* and 1000 grain weight (9% of the variability), and the number of internodes. Moderate negative correlations between the presence of *Ppd-D1a* and the requirements for the sum of active temperatures, active daylight hours, and heading date (40% of the variability) and the number of spikelets (40% of the variability) were also revealed (Table 5, Fig. 2).

Thus, both in Moscow and Krasnodar, aside from the reduction in plant height, *Rht-B1p* showed a negative impact on the 1000 grain weight. The association between the presence of *Rht-B1p* and the grain weight per main spike was moderate in Moscow and weak in Krasnodar. The association between the presence of *Rht-B1p* and the grain number per main spike was weak in both regions of the field experiment. Thus, in Krasnodar the selection for plants of spring wheat that carry *Rht-B1p*, with a slight decrease of 1000 grain weight and grain number per main spike is more likely, as the correlation between the presence of *Rht-B1p* and these characteristics is weak (Table 5, Fig. 2).

**Fig. 3:**
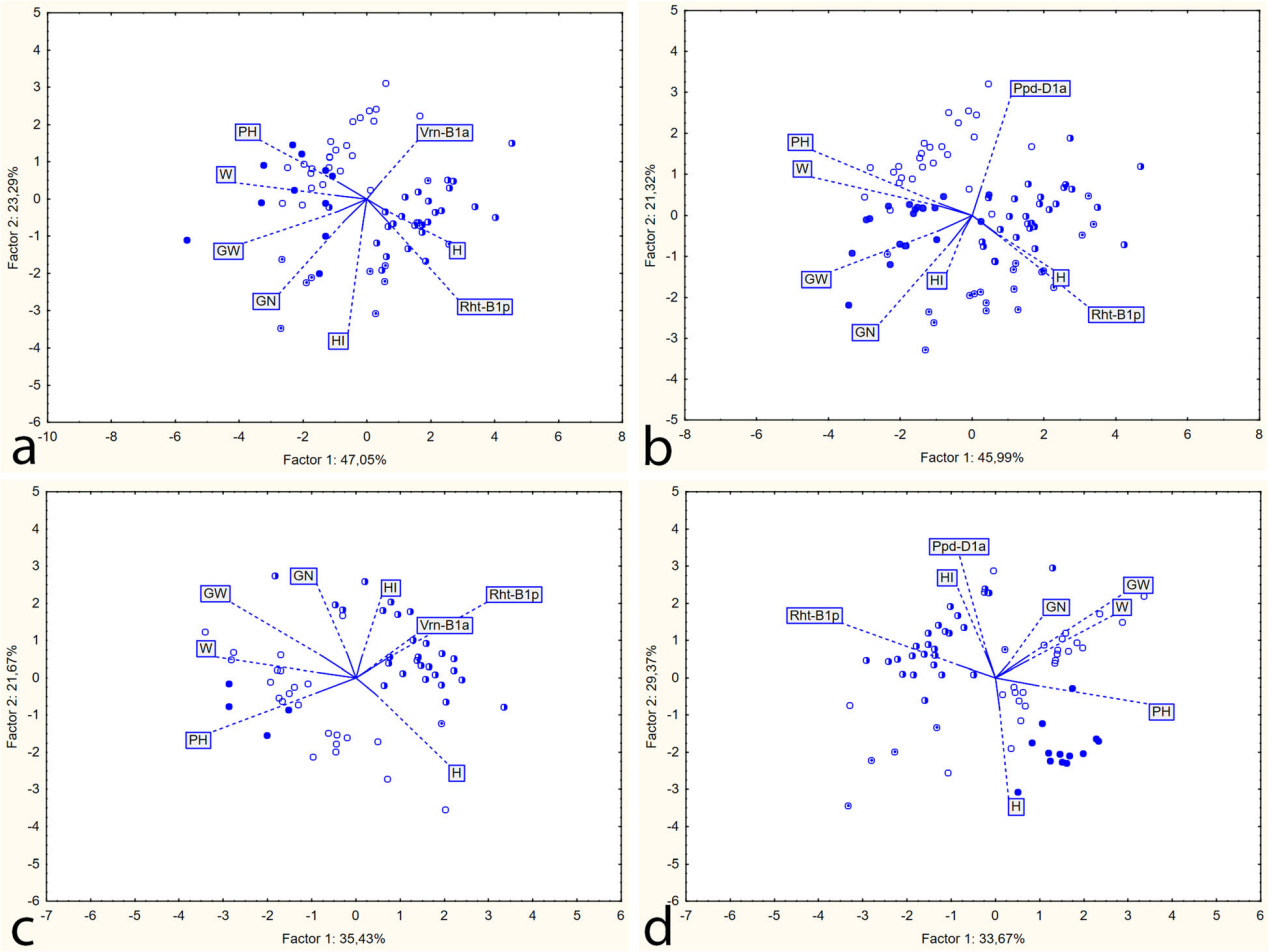
Principal Components Analysis Biplot: а) interaction between *Rht-B1p* and *Vrn-B1a*, Moscow; b) interaction between *Rht-B1p* and *Ppd-D1a*, Moscow; c) interaction between *Rht-B1p* and *Vrn-B1a*, Krasnodar; d) interaction between *Rht-B1p* and *Ppd-D1a*, Krasnodar. Depiction of genotypes of families for a and c are shown as follows: ○, RRVV; ●, RRvv; ◐, rrVV; ⊙, rrvv; depiction of genotypes of families for b и d: ○, RRPP, ●, RRpp; ◐, rrPP, ⊙, rrpp. Vectors are shown in full lines; dashed lines are used for the labels

The Principal Component Analysis (PCA) performed for *Rht-B1p* and *Vrn-B1a* demonstrated the following tendencies (Fig. 3a and c). The *Rht-B1p* and plant height (PH) vectors are directed opposite to each other; “RR” families (empty and filled circles) are located near the PH vector (plant height), which generally describes them as tall plants. Conversely, “rr” families (half-circles and a dot) are in the opposite direction, close to the *Rht-B1p* vector. As can be seen in the biplot in Fig. 3a (Moscow), the circles that are most productive (i.e. close to vectors GW, GN, W) are dark circles (RRvv) and with a dot (rrvv), that is, plants with the recessive *vrn-B1* allele. At the same time, in the biplot for Krasnodar, no tendencies can be observed for the distribution of the circles in relation to the vectors of the productivity elements (Fig. 3c). In addition, no patterns for scattering with respect to the heading date vector (H) were detected for the groups of genotypes. Thus, selection for productive semi-dwarf plants in Moscow is more achievable among plants with *Vrn-A1/vrn-B1* alleles, and in Krasnodar among those with both variants of the *Vrn-A1/vrn-B1* and *Vrn-A1/Vrn-B1a* alleles.

The PCA analysis of the interaction between *Rht-B1p* and the photoperiod-insensitive allele *Ppd-D1b* demonstrated the following trends (Fig. 3b and d): *Rht-B1p* and plant height (PH) vectors are directed opposite to each other; “RR” families (empty and filled circles) are located near the PH vector (plant height), which generally describes them as tall plants. Conversely, “rr” families (half circles and with a dot) are located in the opposite direction, close to the *Rht-B1p* vector*.* As can be seen in the biplot in Fig. 3b, the most productive (i.e. close to vectors GW, GN, W) are dark circles (RRpp) and with rrpp; that is, plants with the sensitivity allele *Ppd-D1b*. However, in the biplot in Fig. 3d (Krasnodar), the most productive families are represented by empty (RRPP) and a half (rrPP) circles; that is, plants with insensitivity *Ppd-D1a*. Thus, it is more likely to select for more productive semi-dwarf plants among the families with the photoperiod-sensitive allele *Ppd-D1b* in Moscow, and with the photoperiod-insensitive allele *Ppd-D1a* in Krasnodar.

## Discussion

The application of new dwarfing alleles in wheat breeding in combination with the alleles of vernalization and photoperiod sensitivity allows for an expansion in the adaptability of this crop to various cultivation conditions. The yield of wheat is to higher extent is determined by the combination of the allelic variants of *Rht*, *Vrn* and *Ppd* reffered to as “core genes” or “adaptaion genes” by some authors [52, 53]. In our research, we evaluated the phenotype of the new dwarfing allele *Rht-B1p* in spring bread wheat in combination with the alleles of vernalization gene *VRN-B1* and the photoperiod-sensitive gene *Ppd-D1* in two regions, Moscow and Krasnodar, which differ in the length of daylight and soil and climatic conditions. In our study, the variation in valuable agronomic traits in semi-dwarf plants bearing the *Rht-B1p* allele associated with the phenotypic manifestation of the *Ppd-D1a* and *Ppd-D1b* alleles was more diverse than that associated with the *Vrn-B1a* and *vrn-B1* alleles. The stronger phenotypic effects of *Ppd-D1* in Krasnodar can be explained by the short daylight conditions in which the effect of the photoperiod-insensitive gene becomes evident. Under the conditions of a long day in Moscow, the influence of the allelic condition of this gene is not noticeable.

Among the GA-insensitive dwarfing alleles of *Rht-B1*, the most common allele is *Rht-B1b*. The effect of *Rht-B1b* on plant height is well studied and varies in the range of 10–25% [14, 15, 27, 54, 55]. In our studies, the difference in height between plants with the allele *Rht-B1p* and *Rht-B1a* ranged between 27 and 34%. This difference in height between *Rht-B1p* and *Rht-B1a* homozygous plants was similar to that obsreved in segragating population in the same cross in a F_2_ vegetative experiment [7], as well as when comparing mutant forms of the Mutant Chris, donor of *Rht-B1p* (previously designted as *Rht17*), with the original wheat variety Chris [19]. Noteworthy, the phenotypic effect of *Rht-B1p* on plant height is comparable to *Rht-B1e*. Analysis of the nucleotide sequences of the alleles showed that stop codons caused by point mutations are found nearby in *Rht-B1e* and *Rht-B1p* [7], which may be the probable reason for the close phenotypic manifestation of these alleles. The *Rht-B1e* allele was detected in 16.5% of accessions from the collection from southern Russia [8]. *Rht-B1p* can be used in breeding in conjunction with *Rht-B1e*, after replacing the negative genetic background of the Chris Mutant.

In our experiments, the *Ppd-D1a* allele in Moscow did not have a statistically significant effect on plant height, although we did note a tendency toward reduced plant height in the experiment in Moscow by 2–5%. In a field experiment in Krasnodar, semi-dwarf plants carrying *Rht-B1p* with the allele *Ppd-D1a* were significantly taller than those with allele *Ppd-D1b* by 12%; that is, the photoperiod-insensitive allele had an effect opposite to the height-reducing effect of *Rht-B1p*. Li et al. (2013) showed that the *Rht-B1h* allele reduced plant height only in plants with the *Ppd-D1a* allele [2]. According to the published data, *Ppd-D1a* reduces height in the range of 4–15% [9, 17, 49–51, 56]. There is an assumption that the effect of *Ppd-D1a* is due to its linkage to the *Rht-8c* dwarfing allele, since this allele also leads to photoperiod insensitivity and to accelerated flowering and heading [50]. However, we have shown that both parent forms carry the wild-type allele *Rht-8a*, which when present in the genome leads to a tall plant phenotype. Therefore, in our opinion, the difference in height under the influence of this allele *Ppd-D1a*, is first of all due to the change in the duration of the vegetative phase, which in turn leads to a change in the habit of the plant.

In our studies in both regions of the field experiment, weak negative correlation between spikelet number per spike and the presence of photoperiod-insensitive allele *Ppd-D1a* in plants and was observed. This tendency agrees with many previous studies [43, 50, 51].

In our studies in Moscow, *Rht-B1p* on average negatively affected grain number per main spike due to a decrease in grain number per spikelet. Moreover, this trait was also influenced by the interaction with the *VRN-B1* gene. In Krasnodar, there was a tendency to an increase in grain number per spike due to an increase in grain number per spikelet and spikelet number per main spike in plants with *Rht-B1p*, but only in the genetic background of *Ppd-D1a* and *Vrn-B1a*. This suggests a multidirectional interaction between the alleles of *Ppd-D1, Rht-B1* and *VRN-B1* genes, depending on the region of the experiment.

In our field experiment in Moscow, a negative influence of the photoperiod-insensitive allele *Ppd-D1a* on the grain number per spike was shown (a decrease of 8–11%, a weak but significant negative correlation). While in Krasnodar, in contrast, grain number per spike was greater in semi-dwarf plants with *Ppd-D1a*. Many studies show that *Ppd-D1* affects 1000-grain weight, vegetative weight, harvest index and grain yield [57, 58]. Alleles of photoperiod sensitivity have both negatively and positively influenced grain number per spike, as well as the percentage of fertile flowers in different experiments [50, 51, 59].

Under the influence of *Rht-B1p*, 1000 grain weight decreased, which is especially noticeable in the PCA biplots where the *Rht-B1p* and 1000 grain weight vectors have opposite directions (Fig. 3). In our experiments, 1000 grain weight decreased under the influence of *Rht-B1p* by 14–18% in Moscow and by 9–21% in Krasnodar when different groups of genotypes were compared. A number of studies have shown that *Rht-B1b* reduces 1000 grain weight (or the weight of individual grains), although in a number of experiments the effect was not statistically significant [14, 15, 27, 55, 60]. With a decrease in plant height, the availability of assimilates to the upper flowers in the spikelet increases, whereas in tall plants, as a rule, they do not produce grain. As a result, the number of grains in the ear increases. There is also another side: this leads to a decrease in 1000 grain weight, the grain becomes smaller due to increasing competition for assimilates. In addition, this may be because the straw itself as a source of the organic matter becomes shorter, generating a deficit for grain filling.

We found that *Vrn-В1а* tends to mitigate the negative effect of *Rht-B1p* on grain number per spike in both regions and 1000 grain weight in Krasnodar. We also demonstrated in the experiments in both regions that *Ppd-D1a* can partially compensate for the negative effect of *Rht-B1p* on 1000 grain weight, for 4–9% in Krasnodar and for 2–5% in Moscow, that is in agrrement with literature [50, 51, 61].

In Moscow and Krasnodar, grain weight per main spike from plants homozygous for *Rht-B1p* was lower compared to that of plants homozygous for *Rht-B1a*. The influence of dwarfing on the productivity of plants or spike depends on a large number of multidirectional factors. In the studies by Chen et al. [50, 51], the maximum grain yield per plant was observed in genotypes of tall plants, although semi-dwarf plants with a combination of a GA-sensitive gene *Rht-5* and photoperiod-insensitive alleles had the same yield as that of tall plants [50]. It should be considered that despite the higher potential yield from tall plants, this is not always achievable, especially with intensive cultivation technology where the risk of lodging is high.

Flowering time is the key factor in the adaptation of plants to various environmental conditions and is critical for avoiding the risks of drought, frost or heat during the reproductive period of development. GA-insensitive short-stem alleles generally do not affect flowering time themselves [12, 15]. Neverthelss, some authors observe some effect of *Rht-B1* region on earliness that might be associated with putative genes located in the vicinity of *Rht-B1* [52, 62]. Later flowering / heading is observed in plants with GA-sensitive dwarfing genes [50, 51, 63]. *Ppd-D1* had a much greater effect in Krasnodar, where *Ppd-D1a* accelerated heading by 5–7 days. It is known from the literature that *Ppd-D1a* accelerates flowering on short days [9, 50, 64, 65], which was also observed in our experiments.

Dwarfing alleles reduce the stem height and thereby allow the spike to carry a heavier load. This in turn improves resistance to lodging and, as a result, allows for an increase in the density of standing through a higher seeding rate, ultimately generating increased grain yield per unit area. In our experiments in both Moscow and Krasnodar, the decrease in plant height in spring wheat due to *Rht-B1p* was very strong (about 30%) and the additional effects of the *VRN-B1* and *Ppd-D1* alleles on plant height (even when statistically significant) do not change the overall effect of the gene. In this regard, the mutual influence of *Rht-B1p, VRN-B1* and *Ppd-D1* on productivity elements is of particular interest.

The negative effects of dwarfing alleles can be overcome by combining them with alleles of vernalization and day length sensitivity. The length of the straw is a function of the rate of development of the plant, which depends on the response of tissues to hormones and environmental signals (day length, temperature). Different combinations of alleles from genes regulating these reactions can be made to fine-tune developmental phases and achieve maximum plant productivity. The strategy of choosing the right combination of genes should be developed for each region individually.

For Moscow, the most promising option for selection of semi-dwarf productive plants will be a combination of *Rht-B1p + Ppd-D1b*. For Krasnodar, a promising option for selection of semi-dwarf productive plants will be a combination of *Rht-B1p + Ppd-D1a*. It follows from the following results: i) with different confidence probability *Ppd-D1a* has a rather negative effect on productivity in Moscow and a positive impact in Krasnodar (the results of the analysis of variance); ii) a weak significant negative correlation in Moscow and positive correlation in Krasnodar between the elements of productivity and *Ppd-D1a* (the results of the regression analysis); and iii) most productive families were in the direction opposite to the *Ppd-D1a* vector in Moscow and in the direction of *Ppd-D1a* in Krasnodar (the results of the PCA analysis).

Differences in the influence of *Rht-B1p* on the grain number per spike in Moscow and Krasnodar can be explained as follows: Moscow is characterized by sod-podzol, humus-poor soils, whereas the soils of Krasnodar are typical chernozems. Probably, the *Rht-B1p* allele studied by us has a positive effect on the number of grains in plants only at high soil fertility. It should be remembered that during the “Green revolution” dwarfing alleles *Rht-B1b* and *Rht-D1b* were introduced in order to increase yield and lodging resistance at high doses of fertilizers, irrigation, and intensive cultivation technology. It is also noteworthy that the positive effects of *Rht-B1p* were manifested in the experiment in Krasnodar only in plants with *Ppd-D1a*.

In its effect on plant height and productivity elements, *Rht-B1p* is close to *Rht-B1e*, which is distributed among 16.5% of varieties in the southern regions of Russia, as well as among some varieties in Ukraine [8, 10, 18]. Thus, *Rht-B1p* can also be used on an equal footing with other dwarfing alleles in the breeding of wheat in the Krasnodar Krai.

Thus, *Rht-B1p* significantly reduces the height of plants, but it can also reduce their productivity. Alleles of genes that regulate developmental type and sensitivity to day length can mitigate unwanted manifestations of *Rht-B1p* in favorable regions. At the same time, in Krasnodar, the interaction of *Rht-B1p* and *Ppd-D1a* genes influencing valuable agronomic traits is most pronounced at short day length.

## Conclusions

In summary, we estimated the effects of *Rht-B1p* on plant height and traits of agronomic value in spring bread wheat in Moscow and Krasnodar. Plant height was reduced on average by 21 cm (28%) and 25 cm (30%), respectively, *Ppd-D1* tended to strengthen the dwarfing effect in Moscow and mitigate it in Krasnodar. Grain weight per main spike was reduced by *Rht-B1p* in Moscow and to lesser extent in Krasnodar. Thousand grain weight was reduced on average by 5.3 g (16%) and 2.9 g (10%) in Moscow and Krasnodar, respectively. The harvest index was increased due to *Rht-B1p* on average by 6 and 10% in Moscow and Krasnodar, respectively. In Krasnodar, *Ppd-D1a* triggered heading 6 days earlier and partially compensated for the loss of grain weight per main spike and thousand grain weight due to *Rht-B1p*. Among semi-dwarf plants with *Rht-B1p,* the most productive were observed among families with *Ppd-D1b* in Moscow and *Ppd-D1a* in Krasnodar. In its effect on plant height and productivity elements, *Rht-B1p* is close to *Rht-B1e,* which is distributed among varieties in the southern regions of Russia and Ukraine. Thus, *Rht-B1p* can be used on an equal footing with other dwarfing alleles in wheat breeding in the Krasnodar Krai.

## Methods

### Plant material

For the initial plant material, we used seeds of the F_2_ population of Chris Mutant/Novosibirskaya 67, kindly provided by Prof. Nobuyoshi Watanabe (College of Agriculture, Ibaraki University) [7]. The seeds of parental plants Chris Mutant (accession number CItr 17,241) and Novosibirskaya 67 (accession number 48601) are available at Germplasm Research International Network and N.I. Vavilov Research Institute of Plant Industry (VIR) (Saint Petersburg, Russia), respectively. The parental plants were analyzed for the following alleles of *Rht*, *VRN*, and *Ppd* in their genotypes: *Rht-B1a* (wild type), *Rht-B1b*, *Rht-B1e*, and *Rht-B1p* for *Rht-B1*; *Rht-D1a* (wild type) and *Rht-D1b* for *Rht-D1*; *Rht-8a* (wild type)/*Rht-8c* for *Rht-8*; *vrn-A1*, *Vrn-A1a*, and *Vrn-A1b* for *VRNA-1*; *vrn-B1*, *Vrn-B1a*, and *Vrn-B1c* for *VRN-B1*; *vrn-D1*, and *Vrn-D1a* for *VRN-D1*; *Ppd-D1a* and *Ppd-D1b* for *Ppd-D1* (see *Molecular analysis* and Table 6). The following allelic states were revealed: Chris Mutant*: Rht-B1p Rht-B1p*\cr*Rht-D1a Rht-D1a*\cr*Rht-8a Rht-8a*\cr*Ppd-D1a Ppd-D1a*\cr*Vrn-A1a Vrn-A1a*\cr*vrn-B1 vrn-B1* \cr*vrn-D1 vrn-D1;* Novosibirskaya 67: *Rht-B1a Rht-B1a*\cr*Rht-D1a Rht-D1a*\cr*Rht-8a Rht-8a*\cr*Ppd-D1b Ppd-D1b*\cr*Vrn-A1a Vrn-A1a*\cr*Vrn-B1a Vrn-B1a*\cr*vrn-D1 vrn-D1.* Therefore, the F_2_ population Chris Mutant/Novosibirskaya 67 segregated in the flowing three loci: *Rht-B1* (*Rht-B1a*/*Rht-B1p*), *Ppd-D1* (*PpdD1a*/*PpdD1b*), and *VRN-B1* (*Vrn-B1a*/*vrn-B1*).

**Table 6:** Description of the molecular markers applied in the study for the identification of the allelic state of *Rht*, *VRN* and *Ppd* genes

Gene	Primer sequence	Allele	Expected size of the product, bp	Reference
*Rht-B1*	Rht-B1p-WF: 5′ ACATGGCGGACGTGGTGC 3′ Rht-B1-R1: 5′ GCCGAGAGAGGACGAT 3’	*Rht-B1a*	425	[7, 66]
	Rht-B1p-F: 5’ ACATGGCGGACGTGGTGT 3′ Rht-B1-R1: 5′ GCCGAGAGAGGACGAT 3’	*Rht-B1p*^a^ (*Rht17*)	425	
	BF: 5’ GGTAGGGAGGCGAGAGGCGAG 3′ WR1: CATCCCCATGGCCATCTCGAGCTG	*Rht-B1a*	237	[67]
	BF: 5′ GGTAGGGAGGCGAGAGGCGAG 3′ MR1: 5′ CATCCCCATGGCCATCTCGAGCTA 3′	*Rht-B1b*^a^ (*Rht1*)	237	
	BF: 5′ GGTAGGGAGGCGAGAGGCGAG 3′ W3: 5′ GGCCATCTCCAGCTGCTCCAGCTT 3’	*Rht-B1a*	228	[66]
	BF: 5’ GGTAGGGAGGCGAGAGGCGAG 3′ M3: 5′ GGCCATCTCCAGCTGCTCCAGCTA 3’	*Rht-B1e*^a^ (*Rht11*)	228	
*Rht-D1*	DF2: 5’- GGCAAGCAAAAGCTTCGCG −3′ WR2: 5’- GGCCATCTCGAGCTGCAC −3’	*Rht-D1a*	264	[67]
	DF: 5’- CGCGCAATTATTGGCCAGAGATAG − 3′ MR2: 5’- CCCCATGGCCATCTCGAGCTGCTA − 3’	*Rht-D1b*^a^ (*Rht2*)	254	
*Ppd-D1*	Ppd-D1_F: ACGCCTCCCACTACACTG Ppd-D1_R2: CACTGGTGGTAGCTGAGATT Ppd-D1_R1: GTTGGTTCAAACAGAGAGC	*Ppd-D1a*	288	[68]
		*Ppd-D1b*^a^	414	
*VRN-A1*	VRN1AF: GAAAGGAAAAATTCTGCTCG VRN1-INT1R: GCAGGAAATCGAAATCGAAG	*Vrn-A1a*	965 + 876	[69, 70]
		*Vrn-A1b*	714	
	VRN1AF: GAAAGGAAAAATTCTGCTCG VRN1-INT1R: GCAGGAAATCGAAATCGAAG	*vrn-A1*^a^	734	
*VRN-B1*	Intr1/B/F: CAAGTGGAACGGTTAGGACA Intr1/B/R3: CTCATGCCAAAAATTGAAGATGA	*Vrn-B1a*	709	[70]
	Intr1: ATCATCTTCTCCACCAAGGG Intr1/B/R3: CTCATGCCAAAAATTGAAGATGA	*Vrn-B1a* *Vrn-B1c*	1124 737	[71]
	Intr1/B/F: CAAGTGGAACGGTTAGGACA Intr1/B/R4: CAAATGAAAAGGAATGAGAGCA	*vrn-B1*^a^	1149	
*VRN-D1*	Intr1/D/F: GTTGTCTGCCTCATCAAATCC Intr1/D/R3: GGTCACTGGTGGTCTGTGC	*Vrn-D1*	1671	[70]
	Intr1/D/F: GTTGTCTGCCTCATCAAATCC Intr1/D/R4: AAATGAAAAGGAACGAGAGCG	*vrn-D1*^a^	997	
*Rht-8*	WMS261-F: 5’- CTCCCTGTACGCCTAAGGC −3′ WMS261-R: 5’- CTCGCGCTACTAGCCATTG − 3′	*Rht-8c*	192	[72]

^a^recessive (or partially recessive) allele

The plants of the F_2_ population of Chris Mutant/Novosibirskaya 67 were planted in pots at 10 seeds per pot and grown in a greenhouse under identical lighting with dosed watering and fertilization. The allelic state of *Rht-B1* was identified for each individual F_2_ plant (see *Molecular analysis* and Table 6). Plants homozygous for *Rht-B1p* and *Rht-B1a* were selected and threshed manually when they achieved complete ripeness. Seeds of F_3_, harvested from individual F_2_ plants, were labelled as a single family. Each family was divided into two equal parts and sown in two climatic regions in field plot tests, in Moscow and Krasnodar. During growth, for each individual F_3_ plant the allelic state of *Rht-B1*, *VRN-B1* и *Ppd-D1* was identified using molecular markers (see *Molecular analysis* and Table 6).

### Molecular analysis

Genomic DNA was extracted from leaves using a CTAB method [73]. PCR was performed in a 25 μL reaction volume, containing 70 mM Tris–HCl buffer (pH 8.6), 16.6 mM (NH4)_2_SO_4_, 2.5 mM MgCl_2_, 0.2 mM of each dNTP, 10%v/v dimethyl sulfoxide, 0.3 μM forward and reverse primers (Sintol Ltd., Moscow, Russia), 1.25 U of Coloured Taq-polymerase (Sileks Ltd., Moscow, Russia) and 100 ng of template DNA. The PCR conditions were as recommended by the authors of the molecular markers (Table 6). The PCR reaction was performed in a GeneAmp PCR System 9700 (Applied Biosystems, Foster City, California, USA). The PCR products were separated in a 1.5% agarose gel in TBE buffer using GeneRuler 100 bp DNA Ladder (Thermo Fisher Scientific, Waltham, Massachusetts, USA) as a molecular weight marker, and stained with ethidium bromide for subsequent visualization in Gel Doc XR+ (Bio-Rad Laboratories, Inc., Hercules, California, USA). The size of the PCR products amplified from molecular marker *Xgwm261*, linked to *Rht-8*, were measured using fragment analysis in a Genetic Analyzer ABI-3130XL (Applied Biosystems, Foster City, California, USA).

### Field experiment

The field experiment was performed in the Field Experimental Station, Russian State Agrarian University—Moscow Timiryazev Agricultural Academy, Moscow (55°50′ N, 37°33′ E, hereinafter – Moscow) and in a plot of land at the National center of grain named after P.P. Lukyanenko in Krasnodar (45°.41′ N, 38°.55′ E, hereafter referred to as Krasnodar) in 2018. The duration of daylight in Moscow was 15:35 at sowing day (May 5), increasing to 17:33 (June 24) and decreasing to 14:49 at the final date of harvesting (August 8); the average daylight duration was 16:40 (long photoperiod). The duration of daylight in Krasnodar was 12:10 at sowing day (March 21) which increased to 15:34 at the final date of harvesting (June 30); the average duration of a day was 14:23 (short photoperiod). Sowing was performed using a breeding cassette drill SKS-6-10 with the following parameters: length of plot 1 m; width of plot, 90 cm; width between the rows, 30 cm (Moscow) or 40 cm (Krasnodar); and distance between the plots, 50 cm. Individual plants were labelled and one leaf from each plant was used for DNA extraction to determine the allelic state of *VRN-B1* and *Ppd-D1,* as well as for verification of the allelic state of *Rht-B1* (see *Molecular analysis* and Table 6). The plots were treated with pesticides to control pests, the weeds were removed manually. Plants of each family were harvested manually at complete ripeness (August 8 in Moscow and June 30 in Krasnodar). The spikes were threshed using a spike thresher MKS-1 M (MZOK Company, Moscow, Russia). The weather conditions in 2018 in Moscow and Krasnodar are displayed in Table 7.

**Table 7:** Temperature and precipitation during the field experiment in Moscow and Krasnodar in 2018

Month	Moscow May 5 – August 19			Krasnodar March 21– June 30		
	Sum of active (> 10 °С) temperatures, °С	Mean monthly temperature, °С	Sum of precipitation, mm	Sum of active (> 10 °С) temperatures, °С	Mean monthly temperature, °С	Sum of precipitation, mm
March	–	–	–	142	6.4	38
April	–	–	–	2845	13.8	26
May	3288	16.1	105	4731	19.4	43
June	3914	17.3	107	5407	24.1	11
July	5097	20.5	190		–	–
August	2926	20.3	39		–	–
Total	15,225	–	441	13,125	–	118

### Phenotyping

In total, 82 families (1035 plants) and 73 families (731 plants) were analyzed in Moscow and Krasnodar, respectively. The following traits were measured in each individual plant: plant height (PH, cm), length of each internode (cm), internode number (IN), main spike length (MSL, cm), spikelet number per main spike (SN), main spike weight (MSW, g), main culm weight (MCW, g), grain weight per main spike (GW, g), and grain number per main spike (GN). The following parameters were calculated for each individual plant: spike compactness (SC, number of spikelets per 10 cm of main spike length), main shoot biomass (MSB, sum of MSW and MCW, g), grain number per spikelet (GNS, GN divided by SN), thousand grain weight (W, thousandfold GWS divided by GNS, g), and harvest index (HI, GW divided by MSB). The heading date was recorded for each family when not less than 80% of the plants came to heading, and based on this date the sum of active (> 10 °C) temperatures to heading date (SAT, ^○^C) and sum of active (> 12 h) light days to heading date (SAD, h) were calculated. The seeds were counted with the use of a Seed Counter [74].

### Statistical analysis

For each phenotypic trait the mean value and confidence interval at a level of significance of 0.01 were calculated. The statistical evaluation of the data was carried out by three one-way ANOVA analyses for each locus (*Rht-B1*, *VRN-B1*, and *Ppd-D1*) and by two two-way ANOVA analyses for the pairwise interaction between loci (*Rht-B1* and *VRN-B1*, *Rht-B1* and *Ppd-D1*). The comparisons between means were detected using a least significant differences (LSD) test at the level of significance of 0.01 and 0.05 (for heading time). Pairwise comparisons of the interaction between *Rht-B1* and *VRN-B1* were performed among families homozygous for the photoperiod-insensitive allele *Ppd-D1а.* Pairwise comparisons of the interaction between *Rht-B1* and *Ppd-D1* were performed among families homozygous for the *Vrn-B1а* allele*.* ANOVA, Correlation and Principal Components Analysis were performed using Statistica 12.0 software (StatSoft, Inc., Tulsa, Oklahoma, USA).

## Supplementary information

**Additional file 1: Table S1.** Mean values of biometric traits for the main shoot in families F_3:4_ Chris Mutant/Novosibirskaya 67 grouped by *Rht-B1*, *VRN-B1*, and *Ppd-D1* alleles. **Table S2.** Mean values of biometric traits of spike productivity traits in families F_3:4_ Chris Mutant/Novosibirskaya 67 grouped by *Rht-B1*, *VRN-B1*, and *Ppd-D1* alleles. **Table S3.** Mean values of harvest index and main spike traits in families F_3:4_ Chris Mutant/Novosibirskaya 67 grouped by *Rht-B1*, *VRN-B1*, and *Ppd-D1* alleles (upper part, one-way NOVA) and by *Rht-B1* × *VRN-B1* and *Rht-B1* × *Ppd-D1* alleles (lower part, two-way ANOVA). **Table S4.** Mean values of heading date, sum of active (> 10 °C) temperatures and active (> 12 h) light days from sowing to heading date in families F_3:4_ Chris Mutant/Novosibirskaya 67 grouped by *Rht-B1*, *VRN-B1*, and *Ppd-D1* alleles. **Table S5.** Mean values of spike parameters in families F_3:4_ Chris Mutant/Novosibirskaya 67 grouped by *Rht-B1*, *VRN-B1*, and *Ppd-D1* alleles (upper part, one-way ANOVA) and by *Rht-B1* × *VRN-B1* and *Rht-B1* × *Ppd-D1* alleles (lower part, two-way ANOVA).

## Data Availability

All data generated or analysed during this study are included in this published article and Additional file 1. The seeds of the parental plant Chris Mutant are available at Germplasm Research International Network (https://npgsweb.ars-grin.gov/gringlobal/search.aspx, accession number CItr 17241); the seeds of the parental plant Novosibirskaya 67 are available at N.I. Vavilov Research Institute of Plant Industry (VIR), Saint Petersburg, Russia (http://db.vir.nw.ru/virdb/maindb, accession number 48601). The studied recombinant isogenic families with different allelic combinations of the allelic state of *Rht-B1* (*Rht-B1a*/*Rht-B1p*), *PpdD1* (*PpdD1a*/*PpdD1b*), and *VRN-B1* (*Vrn-B1a*/*vrn-B1*) loci designated M17/1-М17/81 are being maintained and available upon request from All-Russia Research Institute Of Agricultural Biotechnology (iab@iab.ac.ru, divashuk@gmail.com) and at the National center of grain named after P.P. Lukyanenko (kniish@kniish.ru).

## Abbreviations

GA: Gibberellic acid
R: *Rht-B1a*
r: *Rht-B1p*
V: *Vrn-B1а*
v: *vrn-B1*
P: *Ppd-D1a*
p: *Ppd-D1b*
PH: Plant height
IN: Internode number
MSL: Main spike length
SN: Spikelet number per main spike
MSW: Main spike weight
MCW: Main culm weight
GW: Grain weight per main spike
GN: Grain number per main spike
SC: Spike compactness
MSB: Main shoot biomass
GNS: Grain number per spikelet
W: Thousand grain weight
HI: Harvest index
SAT: Sum of active (> 10 °C) temperatures to heading date
SAD: Sum of active (> 12 h) light days to heading date
