# Supplementary material for: Effects of Rht17 in combination with Vrn-B1 and Ppd-D1 alleles on agronomic traits in wheat in black earth and non-black earth regions
Source: BMC Plant Biol. 2020 Oct 14;20(Suppl 1):304. doi: 10.1186/s12870-020-02514-0 (PMC7556923; doi:10.1186/s12870-020-02514-0)
Supplement: Supplementary file 1 — Additional file 1: Table S1. Mean values of biometric traits for the main shoot in families F3:4 Chris Mutant/Novosibirskaya 67 grouped by Rht-B1, VRN-B1, and Ppd-D1 alleles. Table S2. Mean values of biometric traits of spike productivity traits in families F3:4 Chris Mutant/Novosibirskaya 67 grouped by Rht-B1, VRN-B1, and Ppd-D1 alleles. Table S3. Mean values of harvest index and main spike traits in families F3:4 Chris Mutant/Novosibirskaya 67 grouped by Rht-B1, VRN-B1, and Ppd-D1 alleles (upper part, one-way NOVA) and by Rht-B1 × VRN-B1 and Rht-B1 × Ppd-D1 alleles (lower part, two-way ANOVA). Table S4. Mean values of heading date, sum of active (> 10 °C) temperatures and active (> 12 h) light days from sowing to heading date in families F3:4 Chris Mutant/Novosibirskaya 67 grouped by Rht-B1, VRN-B1, and Ppd-D1 alleles. Table S5. Mean values of spike parameters in families F3:4 Chris Mutant/Novosibirskaya 67 grouped by Rht-B1, VRN-B1, and Ppd-D1 alleles (upper part, one-way ANOVA) and by Rht-B1 × VRN-B1 and Rht-B1 × Ppd-D1 alleles (lower part, two-way ANOVA). [file 12870_2020_2514_MOESM1_ESM.docx]

Table S1. Mean values of biometric traits for the main shoot in families F_2:4_ Chris Mutant/Novosibirskaya 67 grouped by *Rht-B1*, *VRN-B1*, and *Ppd-D1* alleles

| Genotype | Plant height, cm | | Peduncle length, cm | | Main shoot biomass, g | |
| --- | --- | --- | --- | --- | --- | --- |
|  | Moscow | Krasnodar | Moscow | Krasnodar | Moscow | Krasnodar |
| RR | 73.9±1.4a^*^ | 84.0±0.9a | 33.1±1.0a | 36.1±0.6a | 2.3±0.1a | 3.6±0.1a |
| rr | 53.3±1.3b | 58.8±1.2b | 22.5±0.9b | 24.0±0.5b | 1.8±0.1b | 3.1±0.3b |
| VV | 63.4±1.0a | 71.5±0.8a | 27.8±0.7a | 30.0±0.4a | 2.0±0.1a | 3.4±0.1a |
| vv | 64.4±2.7a | 69.4±3.6a | 27.8±1.9a | 29.9±2.1a | 2.1±0.2a | 3.1±0.7a |
| PP | 63.4±1.0b | 71.5±0.7b | 27.8±0.7a | 30.0±0.4b | 2.0±0.1b | 3.4±0.1a |
| pp | 65.4±1.6a | 68.9±1.4a | 29.0±1.1a | 28.3±0.8a | 2.2±0.1a | 3.3±0.3a |

Mean value± confidence intervals at 0.01 significance level are shown.

*Mean values designated with the same letters have no significant differences within each group of two genotypes (RR and rr; VV and vv; PP and pp) as calculated using one-way ANOVA.

Table S2. Mean values of biometric traits of spike productivity traits in families F_2:4_ Chris Mutant/Novosibirskaya 67 grouped by *Rht-B1*, *VRN-B1*, and *Ppd-D1* alleles.

| Genotype | Grain weight in main spike, g | | Grain number per main spike, pcs. | | Thousand grain weight, g | |
| --- | --- | --- | --- | --- | --- | --- |
|  | Moscow | Krasnodar | Moscow | Krasnodar | Moscow | Krasnodar |
| RR | 1.18±0.05a^*^ | 1.44±0.05a | 35.5±1.2a | 48.5±1.4a | 32.9±0.7a | 29.8±0.7a |
| rr | 0.94±0.05b | 1.35±0.05b | 33.3±1.5a | 50.0±1.4a | 27.6±0.8b | 26.9±0.7b |
| VV | 1.06±0.04a | 1.39±0.04a | 34.4±1.0a | 49.3±1.0a | 30.2±0.5a | 28.3±0.5b |
| vv | 1.08±0.11a | 1.29±0.18a | 34.6±2.7a | 45.6±5.0a | 30.5±1.5a | 28.1±2.5a |
| PP | 1.06±0.04b | 1.39±0.04a | 34.4±1.0b | 49.3±1.0a | 30.2±0.6a | 28.3±0.5a |
| pp | 1.12±0.06a | 1.31±0.07b | 37.8±1.6a | 48.5±1.9a | 29.2±0.9b | 26.6±1.0b |

Mean value± confidence intervals at 0.01 significance level are shown.

*Mean values designated with the same letters have no significant differences within each group of two genotypes (RR and rr; VV and vv; PP and pp) as calculated using one-way ANOVA.

Table S3. Mean values of harvest index and main spike traits in families F_2:4_ Chris Mutant/Novosibirskaya 67 grouped by *Rht-B1*, *VRN-B1*, and *Ppd-D1* alleles (upper part, one-way ANOVA) and by *Rht-B1*×*VRN-B1* and *Rht-B1*×*Ppd-D1* alleles (lower part, two-way ANOVA).

| Genotype | Harvest index | | Spikelet number per main spike, pcs | | Grain number per spikelet, pcs | |
| --- | --- | --- | --- | --- | --- | --- |
|  | Moscow | Krasnodar | Moscow | Krasnodar | Moscow | Krasnodar |
| One-way ANOVA | | | | | | |
| RR | 0.50±0.01b^*^ | 0.40±0.01b | 14.5±0.3a | 18.2±0.3a | 2.5±0.1a | 2.7±0.1b |
| rr | 0.53±0.02a | 0.44±0.01a | 14.5±0.2a | 17.9±0.3a | 2.3±0.1a | 2.8±0.1a |
| VV | 0.52±0.01a | 0.42±0.01a | 14.4±0.2a | 18.0±0.2a | 2.4±0.1a | 2.7±0.1a |
| vv | 0.50±0.03a | 0.42±0.03a | 14.7±0.5a | 18.2±1.1a | 2.4±0.2a | 2.5±0.3a |
| PP | 0.52±0.01a | 0.42±0.01a | 14.4±0.2b | 18.0±0.2b | 2.4±0.1b | 2.7±0.1a |
| pp | 0.51±0.01a | 0.39±0.01b | 15.1±0.3a | 19.3±0.4a | 2.5±0.1a | 2.5±0.1b |
| Two-way ANOVA | | | | | | |
| RRVV | 0.51±0.01b | 0.40±0.01b | 14.5±0.3a | 18.1±0.3a | 2.4±0.1ab | 2.7±0.1b |
| RRvv | 0.49±0.03b | 0.40±0.01b | 14.7±0.6a | 18.3±0.6a | 2.6±0.2a | 2.7±0.2ab |
| rrVV | 0.53±0.02a | 0.45±0.01a | 14.4±0.2a | 17.9±0.3a | 2.3±0.1bc | 2.8±0.1a |
| rrvv | 0.52±0.04ab | 0.43±0.06ab | 14.6±0.8a | 18.0±1.8a | 2.1±0.3c | 2.4±0.6ab |
| RRPP | 0.51±0.01b | 0.42±0.05ab | 14.5±0.3b | 18.1±0.3b | 2.4±0.1ab | 2.7±0.1b |
| RRpp | 0.51±0.01b | 0.37±0.01b | 15.0±0.3a | 19.1±0.3a | 2.5±0.1a | 2.6±0.1bc |
| rrPP | 0.53±0.02a | 0.45±0.01a | 14.4±0.2b | 17.9±0.3b | 2.3±0.1b | 2.8±0.1a |
| rrpp | 0.52±0.03ab | 0.40±0.02ab | 15.2±0.5a | 19.5±0.5a | 2.5±0.2ab | 2.5±0.2c |

Mean value± confidence intervals at 0.01 significance level are shown.

*Mean values designated with the same letters have no significant differences within each group of two genotypes (RR and rr; VV and vv; PP and pp) as calculated using one-way ANOVA or each group of four genotypes (RRVV, RRvv, rrVV, rrvv and RRPP, RRpp, rrPP, rrpp) as calculated using two-way ANOVA.

Table S4. Mean values of heading date, sum of active (>10 ^○^C) temperatures and active (>12 hours) light days from sowing to heading date in families F_2:4_ Chris Mutant/Novosibirskaya 67 grouped by *Rht-B1*, *VRN-B1*, and *Ppd-D1* alleles

| Genotype | Heading date, days from sowing to heading | | Sum of active (>10 ^○^C) temperatures to heading date, ^○^C | | Sum of active (>12 hours) light days to heading date, h | |
| --- | --- | --- | --- | --- | --- | --- |
|  | Moscow | Krasnodar | Moscow | Krasnodar | Moscow | Krasnodar |
| RR | 53.0±0.5b^*^ | 64.6±1.5a | 816±10b | 853±32a | 880±8b | 877±23a |
| rr | 54.4±0.6a | 64.7±0.9a | 845±13a | 855±20a | 904±11a | 878±14a |
| VV | 53.6±0.5a | 64.8±0.9a | 828±10a | 858±19a | 890±8a | 880±14a |
| vv | 53.9±0.8a | 64.1±4.9a | 835±16a | 843±103a | 895±13a | 869±74a |
| PP | 53.6±0.5b | 64.8±1.0b | 828±10b | 858±20b | 890±8b | 880±15b |
| pp | 54.4±0.5a | 71.0±1.9a | 846±11a | 985±40a | 905±9a | 974±29a |

Mean value± confidence intervals at 0.01 significance level are shown.

*Mean values designated with the same letters have no significant differences within each group of two genotypes (RR and rr; VV and vv; PP and pp) as calculated using one-way ANOVA.

Table S5. Mean values of spike parameters in families F_2:4_ Chris Mutant/Novosibirskaya 67 grouped by *Rht-B1*, *VRN-B1*, and *Ppd-D1* alleles (upper part, one-way ANOVA) and by *Rht-B1*×*VRN-B1* and *Rht-B1*×*Ppd-D1* alleles (lower part, two-way ANOVA).

| Genotype | Main spike length, cm | | Spike compactness | |
| --- | --- | --- | --- | --- |
|  | Moscow | Krasnodar | Moscow | Krasnodar |
| One-way ANOVA | | | | |
| RR | 7.7±0.1a | 9.9±0.2a | 19.1±0.3b | 18.5±0.3a |
| rr | 7.3±0.1b | 9.7±0.2a | 19.9±0.3a | 18.5±0.4a |
| VV | 7.5±0.1a | 9.8±0.1a | 19.5±0.3a | 18.4±0.2b |
| vv | 7.5±0.3a | 9.3±0.6a | 19.7±0.7a | 19.7±1.2a |
| PP | 7.5±0.1a | 9.8±0.1a | 19.5±0.3b | 18.4±0.3b |
| pp | 7.6±0.2a | 9.8±0.2a | 20.1±0.4a | 19.8±0.5a |
| Two-way ANOVA | | | | |
| RRVV | 7.7±0.1a | 10.0±0.2a | 19.0±0.4b | 18.4±0.4b |
| RRvv | 7.7±0.4ab | 9.4±0.3b | 19.5±1.0ab | 19.5±0.9a |
| rrVV | 7.3±0.1b | 9.7±0.2ab | 19.9±0.4a | 18.5±0.3b |
| rrvv | 7.4±0.4ab | 9.1±1.2ab | 19.8±1.0ab | 19.9±3.0ab |
| RRPP | 7.7±0.1a | 10.0±0.2ab | 19.0±0.4b | 18.4±0.4c |
| RRpp | 7.6±0.2a | 10.0±0.2a | 19.9±0.4a | 19.2±0.5b |
| rrPP | 7.3±0.1b | 9.7±0.2b | 19.9±0.4a | 18.5±0.4c |
| rrpp | 7.5±0.3ab | 9.7±0.4ab | 20.3±0.7a | 20.4±0.9a |

Mean value± confidence intervals at 0.01 significance level are shown.

*Mean values designated with the same letters have no significant differences within each group of two genotypes (RR and rr; VV and vv; PP and pp) as calculated using one-way ANOVA or each group of four genotypes (RRVV, RRvv, rrVV, rrvv and RRPP, RRpp, rrPP, rrpp) as calculated using two-way ANOVA.
